# Evaluation the Potential of Onion/Laponite Composites Films for Sustainable Food Packaging with Enhanced UV Protection and Antioxidant Capacity

**DOI:** 10.3390/molecules28196829

**Published:** 2023-09-27

**Authors:** Maciel L. Barbosa, Leticia M. de Oliveira, Robert Paiva, Alessandra C. Dametto, Diogenes dos S. Dias, Clovis A. Ribeiro, Magdalena Wrona, Cristina Nerín, Hernane da S. Barud, Sandra A. Cruz

**Affiliations:** 1 Department of Chemistry, Federal University of São Carlos (UFSCar), São Carlos 13565-905, Brazil; maciellb@estudante.ufscar.br (M.L.B.); robert21sp@gmail.com (R.P.); 2 Department of Physics, Federal University of the São Francisco Valley (UNIVASF), Petrolina 56300-000, Brazil; leticia.maria@univasf.edu.br; 3 BioSmart Nanotechnology Ltda., Araraquara 14808-162, Brazil; alessandradametto@gmail.com (A.C.D.); digignes@gmail.com (D.d.S.D.); 4 Institute of Chemistry, São Paulo State University (UNESP), Araraquara 14800-900, Brazil; clovisaugustoribeiro@gmail.com; 5 Engineering Research Institute of Aragon (I3A), University of Zaragoza, María de Luna 3, 50018 Zaragoza, Spain; magdalenka.wrona@gmail.com; 6 Laboratory of Biopolymers and Biomaterials (BIOPOLMAT), University of Araraquara (UNIARA), Araraquara 14801-320, Brazil; hernane.barud@gmail.com

**Keywords:** biopolymer, onion films, composite, laponite, antioxidant

## Abstract

Agro-industrial residues have attracted attention for their applications in the field of biodegradable packaging. Recently, our research group has developed onion-based films with promising properties for this type of application due to their non-toxicity, biocompatibility and biodegradability. Therefore, in this study, we investigated the effect of Laponite clay concentration on the physicochemical and antioxidant properties of the onion-based films, which were prepared by a casting method. The XRD and FTIR data confirm the presence of the mineral clay in the onion-based films. These findings are consistent with those obtained from FE-SEM analysis, which revealed the presence of typical Laponite grains. In terms of wettability, the results show that the clay decreases the hydrophilic character of the material but slightly increases the water vapor permeation. Optical characterization revealed that the materials exhibited zero transmittance in the UV region and increased opacity in the visible region for composites containing 5% and 10% Laponite. Furthermore, the antioxidant test demonstrated higher antioxidant potential in the composites compared to the pure films. Consequently, these results suggest that the formation of Laponite and onion composites could be an essential strategy for developing natural polymers in the field of food contact packaging.

## 1. Introduction

Onions are widely used as a seasoning vegetable worldwide, originally originating in Central Asia, and are known for their various health properties [1]. They consist of soluble and non-structural carbohydrates (fructooligosaccharides and polysaccharides), thiosulfinates, and flavonoids [2,3]. The primary cell walls of onions have cellulose, hemicelluloses, and pectins as the main structural carbohydrates [4,5].

In the last 10 years, global onion production has increased by at least 25%, making it the second most important horticultural crop, with a current production of around 83 million tons [6]. This has led to an increase in demand for processed onions, generating a substantial amount of antioxidant-rich industrial waste. These residues pose an environmental challenge. However, they can be utilized to minimize waste and mitigate environmental harm, aligning with current industry trends and the principles of a circular economy and sustainability [7,8].

Onions are known as one of the most common sources of flavonoids in the human diet [6], one of the most important is quercetin, which not only possesses beneficial effects against several pathologies but also offers a wide range of applications due to its antioxidant and antimicrobial properties, food safety, sustainability, and versatility [9]. According to Teshika et al. [10], onions have significant potential for application in emerging areas of interest, including medical, pharmacological, and food-related fields.

Recently, Dias et al. [11] prepared semi-transparent, flexible films based on *Allium cepa* L. (onion), exhibiting thermal, mechanical, and biocompatibility properties suitable for packaging applications. The films were prepared by a green method, specifically in an aqueous medium, without the addition of solvents or the incorporation of functional additives in the film formulation. In parallel, Barreto et al. [12] observed the absence of mutagenicity and cytotoxicity in these bioplastics. Additionally, Soares et al. [13] obtained positive results in sensory properties, quality, and extended shelf life of beef hamburgers when using this onion-based material as edible packaging.

These studies also suggest that onion-based films have interesting antioxidant properties attributed to their high concentration of flavonoids, which is of paramount importance for food packaging applications. Oxidation is a significant factor contributing to the decline in food quality, affecting nutritional content, sensory attributes, and food safety [14,15]. Consequently, the industry is looking for materials that can mitigate these effects. However, the addition of synthetic and natural antioxidants, as well as the formation of composites, can further enhance these properties.

Currently, the frequent use of mineral clays in combination with biopolymers has demonstrated promising results by improving thermal, mechanical, barrier, and antioxidant properties. Laponite, a synthetic smectite clay, consists of lamellae with a porous structure composed of Mg^2+^ ions in octahedral positions, Li^+^ ions, and Na^+^ ions located in the interlayer regions [16,17].

This clay is already being used as a modifier in biopolymers for packaging applications. For example, Valencia et al. [18] studied the effect of Laponite concentration on some physical properties of gelatin films and observed an improvement in mechanical properties while maintaining water vapor permeation properties. Meanwhile, Li et al. [19] studied the effect of Laponite concentration on the physicochemical properties of gelatin films. The results indicate that with an increase in clay concentration, there is a significant improvement in mechanical properties and water vapor permeability. Moreover, the films showed a significant increase in meat quality during storage, which correlated with the mineral clay concentration.

Some studies with montmorillonite and halloysite clays have shown significant changes in antioxidant properties when incorporated into biopolymer materials [20,21]. However, based on our research, there are no existing reports in the literature regarding the use of Laponite clay as a natural biopolymer modifier to enhance the antioxidant properties of materials when applied to food packaging. Thus, the aim of this study was to incorporate Laponite clay into onion-based films and to evaluate the effect of the formation of this hybrid material on the antioxidant and physicochemical properties. For this purpose, physicochemical characterizations were performed on the pristine and modified films. Additionally, antioxidant capacity tests were performed on both materials and compared with polyethylene tests.

## 2. Results and Discussion

### 2.1. Physicochemical Properties

**Figure 1:**
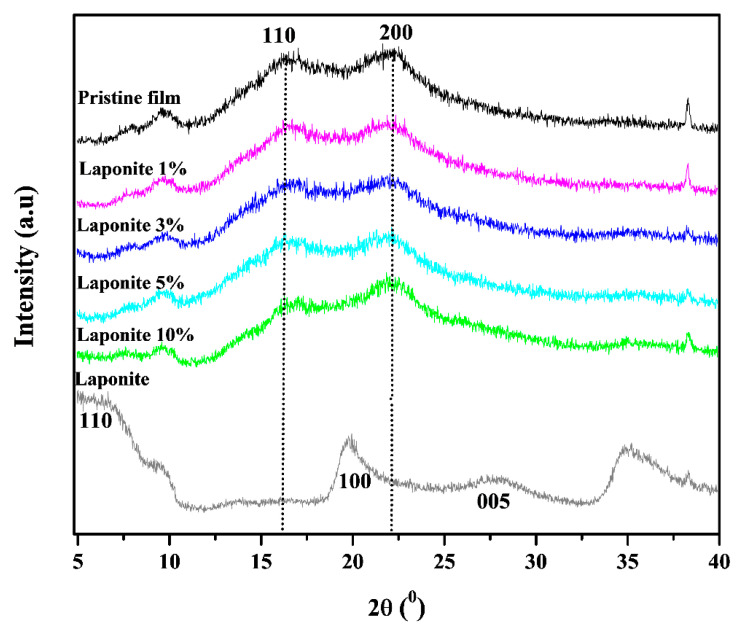
X-ray diffractograms (XRD) pristine film and composite onion/Laponite.

The pristine films and the composites were characterized by X-ray diffractometry (XRD) to evaluate the structure of the material as well as to determine the crystallinity content. Figure 1 presents the XRD for the pristine, onion-based films and all composites.

The film is a semicrystalline polymer, with crystalline and amorphous regions. It is possible to observe that both the pristine film and the composites present broad bands at 16.5° and 22.0° that characterize the crystallographic planes (110) and (200), respectively, of the cellulose crystalline phase present in the onion structure (Dias et al.) [10]. The band at 16.5° suggests, for type I cellulose, the presence of an overlap between triclinic and monoclinic unit cells, while the second refers to the distance between hydrogen bridges in this unit [22].

Note that with increasing clay concentration there is a decrease in the intensity of the characteristic cellulose bands, probably due to the contribution of the intense Laponite peak located at the 20° theta, which corresponds to plan (100) [23]. One can also observe the presence of the main Laponite peaks around 6.3° and 27°, which are characteristic of (001) and (005) planes of the mineral clay [24].

**Figure 2:**
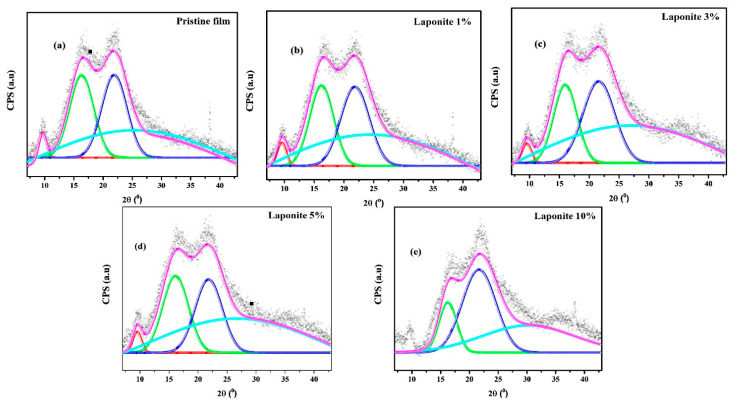
Deconvoluted peaks X-ray diffractograms (XRD) pristine film and onion/Laponite composite: (**a**) Pristine film, (**b**) Laponite 1%, (**c**) Laponite 3%, (**d**) Laponite 5% and (**e**) Laponite 10% **.** The deconvoluted bands are shown in colour for better visualization.

From the deconvolution of the bands, Figure 2, making a relation of the amorphous and crystalline halos, it was possible to calculate the crystallinity index of the films from Equation (3), which is presented in Table 1.

It is possible to observe that with an increasing Laponite concentration there is an increase in the degree of crystallinity. This indicates the presence of the clay in the polymer matrix, which should provide a more organized rearrangement of the polymer chains [16] favoring the formation of crystalline domains, especially at concentrations of 5% and 10%. This type of heterogeneous crystallization has been widely described in the literature [18,25].

**Figure 3:**
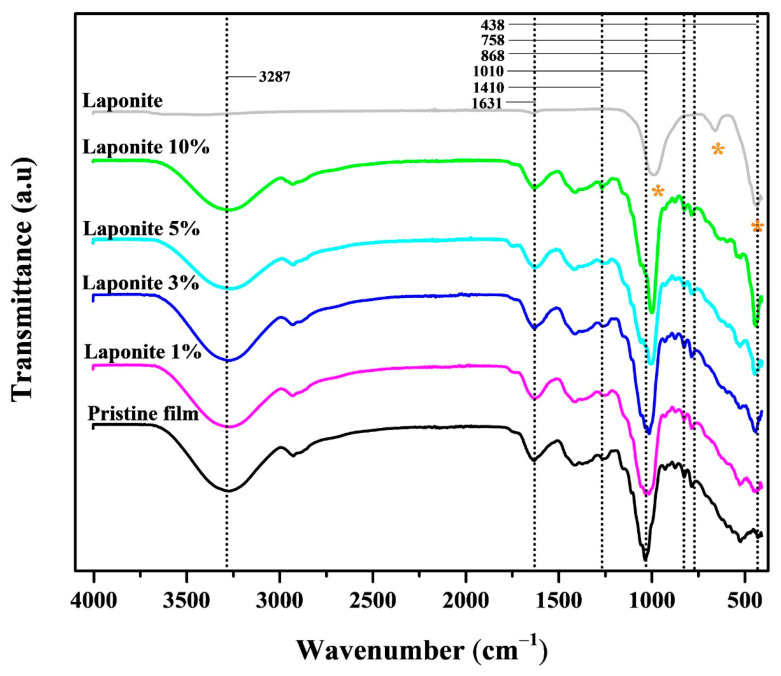
FTIR spectra of the pristine film and composites onion/Laponite. “*”refers to the characteristic peaks of Laponite.

Figure 3 shows the FTIR spectra of both the Laponite and the pristine and modified films. In the case of the clay, a band at 868 cm^−1^ is observed. Characteristic bands of Laponite have been identified around 970 cm^−1^, related to the stretching vibrations of the Si-O and Si-O-Si bonds, and at 442 cm^−1^ (stretching vibration of the Mg-O bond) [26,27].

Absorption bands around 1010 cm^−1^ are typical for unconjugated C-N bonds in primary, secondary, and tertiary amines [28]. Abboud et al. [29] found a band at 1406 cm^−1^, which was attributed to asymmetric CH_3_ deformation. These authors also observed a small band at 1253 cm^−1^ related to a type of amide III.

Another band at 1631 cm^−1^ was identified, (HOH) of absorbed water in pectin and cellulose [30]. Bands between the wavenumbers 1800–750 cm^−1^ reflect mainly carbohydrates, lipids, secondary structures of proteins, and polyphenols in plants [28]. Lu et al. [31] identified a peak at 1618 cm^−1^ related to the C-C of the phenyl ring, which is present at high levels in polyphenolic compounds from *Allium*-type plants.

**Figure 4:**
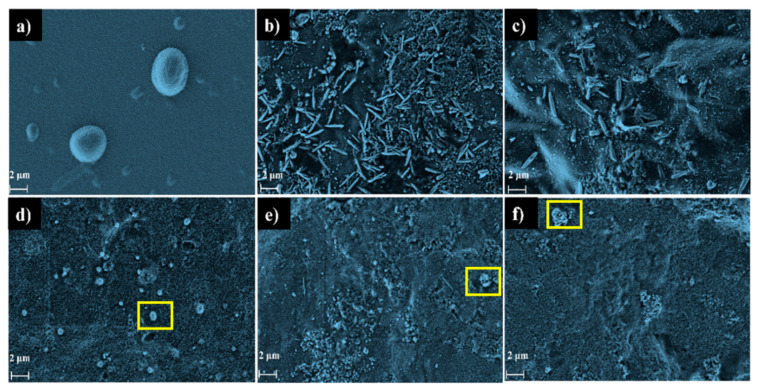
FE-SEM images of the surface of the (**a**) Laponite, (**b**) Pristine film, (**c**) Laponite 1%, (**d**) Laponite 3%, (**e**) Laponite 5% and (**f**) Laponite 10%.

The pristine, modified and Laponite films were analyzed for their morphological aspects by FE-SEM, where it was possible to observe an irregular surface morphology for all materials, as shown in Figure 4a–f. Laponite presents relatively rough grains of irregular size distributed over the surface. Onion films, on the other hand, show elongated fibrils of non-uniform size dispersed over the surface, like the results obtained by Dias et al. [10]. However, with an increasing clay concentration, a more compact surface is observed with the presence of agglomerated grains similar to Laponite (as shown in the highlight) indicating a possible non-uniform dispersion over the surface of the onion-based films.

**Figure 5:**
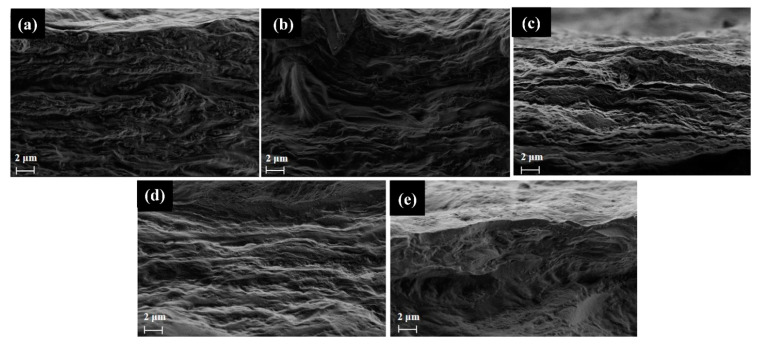
FEG-SEM images of the cross-section. (**a**) Pristine film, (**b**) Laponite 1%, (**c**) Laponite 3%, (**d**) Laponite 5%, and (**e**) Laponite 10%.

The cryogenically fractured cross-section of the films was also evaluated (Figure 5a–e). It can be observed that the pristine and modified materials exhibit a dense, layered, voided structure (unchanged by the presence of clay) typical for onion-based films. A previous study by Dias et al. [10] indicated that the presence of carbohydrates, typical of this material, is responsible for the plasticizing effect that can lead to an increase in the hollow regions.

### 2.2. Surface Wettability Measurements

**Figure 6:**
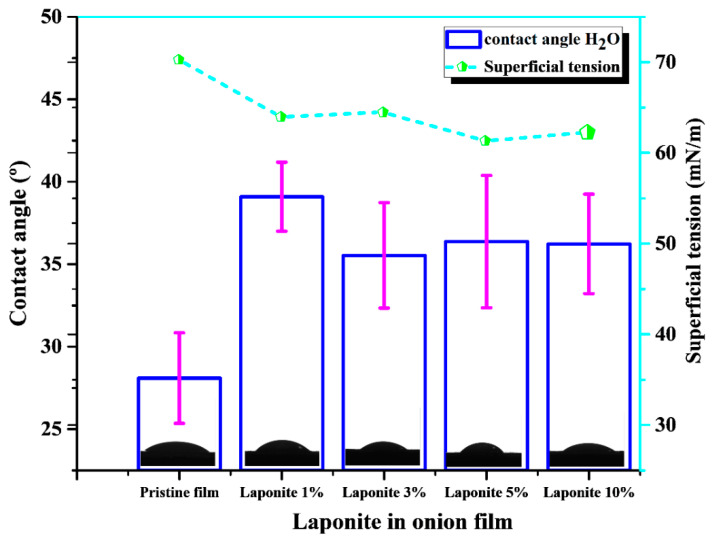
Contact angle and surface tension of pristine and onion/Laponite composites films.

The contact angle and surface tension obtained for the composite materials are shown in Figure 6. For the pristine material, an angle of 28° was obtained, indicating a very hydrophilic surface. This behavior is mainly due to the presence of soluble carbohydrates in the chemical structure of the onion-based films, which are generally hydrophilic compounds (carbohydrates) that tend to interact with water [10,32].

The addition of Laponite slightly increases the contact angle values (35–40°), and consequently the hydrophobicity. This is probably because the presence of the clay reduces the interaction of water with the abundant hydroxyl groups (soluble carbohydrates) present in the biopolymer matrix [33,34]. A second fact could be the possible point formation of hydrophobic groups (e.g., Si-N), as observed in the study of Rouf et al. [35]. Despite this increase in contact angle with the addition of Laponite when compared to the pristine polymer, no significant difference are observed in these properties mainly for the samples containing 5% and 10% Laponite.

It is observed that as the Laponite concentration in the onion-based films increases, the surface tension decreases. This is probably due to the reduced wettability with the addition of the clay, as explained earlier in the contact angle results. When this occurs, the surface energy is weaker than the surface tension of the liquid, which means that the liquid can better maintain its droplet shape. Then, the interfacial tension between the solid and the liquid decreases, because the interaction between the two is not as strong [36].

### 2.3. Water Vapor Permeability

**Figure 7:**
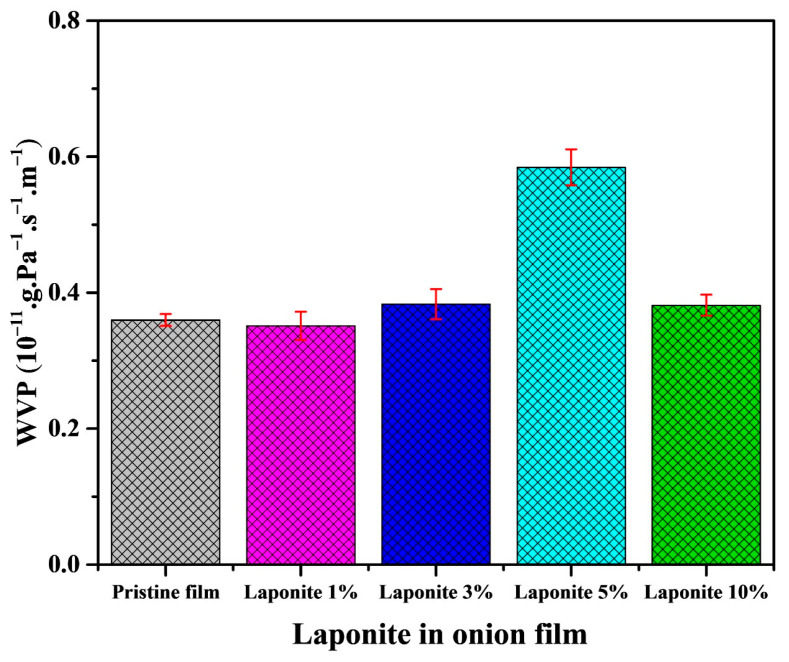
Water vapor permeation in pristine film and onion/Laponite composites.

Water vapor permeability (WVP) is an indicator of the inherent moisture barrier properties of a material and is a critical performance metric in the industrial sector. This includes food packaging and pharmaceutical products, as well as water filtration membranes. Figure 7 shows the WVP results of onion-based films with different concentrations of the Laponite clay.

It is possible to observe that the presence of Laponite in the onion-based films does not interfere so effectively with the WVP values and no significant differences were observed between the samples. However, a tendency to increase these values with the presence of clay is still observed, especially for the sample containing 5% of Laponite. This is probably due to the low interaction of the clay with the biopolymer, as observed by the formation of aggregates in the microscopic surface images and by the presence of empty spaces in the bulk of the material (Figure 5). This low interaction may lead to increased intermolecular free space, which facilitates the passage of water vapor and increases its permeation [37].

This is not the first time that such a fact has been observed, in the work of Silva et al. [17] in which they prepared nanocomposite films based on Laponite and cellulose nanofibers, it is noted that the increase in clay in the nanofibers also increased the WVP values. One of their justifications is based on the way the Laponite is dispersed in the biopolymer matrix. This means that low clay interaction can promote the aggregate formation, resulting in poor dispersion and consequently low tortuosity.

### 2.4. UV-Vis Spectroscopy

**Figure 8:**
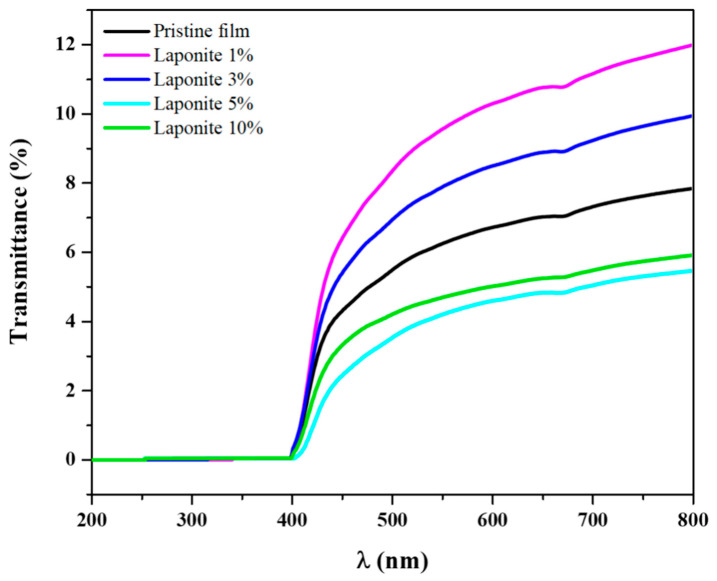
Transmittance spectrum of pristine films and onion/Laponite composites.

Ultraviolet-visible spectroscopy is an important technique door analyzing the light absorption and light transmission properties of films applied to food packaging since light is one of the main factors responsible for reducing the shelf life of food. The pristine onion-based and Laponite-modified films were characterized by transmittance in the 200–800 nm range (Figure 8).

In the visible region, the onion-based film showed a maximum transmittance of 6%, at 550 nm. The composites modified with 1% and 3% had maximum transmittances of 9% and 8%, respectively. However, at concentrations of 5% and 10%, the transmittances are lower than those observed for the pristine film, which is around 4% for both.

To complement the data obtained from the transmittance, the opacity of the films was also calculated using Equation (5). For the pristine film, an opacity value around 7.7 was obtained. The films modified with 1% and 3% of Laponite were slightly more transparent, with a calculated opacity of about 6.5 and 7.5, respectively. However, more opaque films were obtained at concentrations of 5% and 10%, with values close to 9.0 and 9.1, respectively, as shown in Table 1.

As previously presented, the presence of Laponite interfered with the values of transmittance and opacity values of the biopolymer films. Laponite, due to its mineral and structure, can give more transparency to the composites, which was observed at concentrations of 1% and 3%. However, increasing the concentration of Laponite by 5% and 10% in the biopolymer matrix can generate light scattering due to the higher solids content, associated with agglomeration, reducing the transparency, and increasing the opacity of the films. This can be seen in the FE-SEM results (Figure 4), which show the presence of small agglomerates of the clay on the surface of the polymer matrix [38]. These data are of great importance for food packaging applications, since the opaquer the bioplastic is, the less light will pass through it, and consequently, the less the food will be affected by photo-oxidation processes.

Looking at the UV region of the transmittance spectrum, all the films have values close to zero, which means that the passage of this type of radiation is blocked. UV radiation is a significant factor that can contribute to the deterioration of foods, particularly affecting their organoleptic properties and shelf life [39,40].

### 2.5. Antioxidant Properties

**Figure 9:**
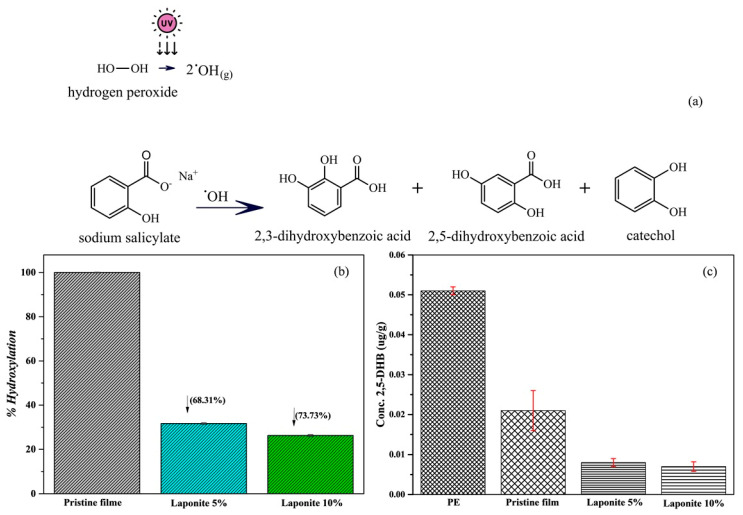
(**a**) Mechanism of sodium salicylate oxidation by UV light irradiation. (**b**) Antioxidant capacity of pristine films and onion/Laponite composites expressed in percentage of hydroxylation. (**c**) Quantification and comparison of 2,5-DHB concentration for PE, pristine film, and composites with 5% and 10% (*m*/*m*) of Laponite.

As described earlier in the methodology section, SA was used to trap OH• radicals, as shown in the reaction mechanism in Figure 9a. Zanta et al. [41] described that the reaction likely occurs via the intermediate hydroxycyclohexadienyl radical, which is activated at the ortho and para positions of the ring. Thus, the fluorescent product of this reaction 2,5-DHB was used for the analysis of the antioxidant capacity of onion films without and with Laponite.

Figure 9b shows the relationship between the percentage of hydroxylation after 24 h and the percentage of Laponite added to the onion-based films, which is characteristic for the formation of the reaction product 2,5-DHB. As can be observed, the addition of Laponite to the onion-based films decreases the percentage of hydroxylation. Therefore, it increases the antioxidant power of the samples and reduces the formation of the fluorescent compound 2,5-DHB, compared to the pristine onion-based film.

As described in some papers, Laponite has a structure composed of crystalline silicate layers with nanometer-sized pores [41,42]. Moreover, their structure and the presence of charges from sodium and magnesium ions can act as scavengers of radicals, charges, particles, and organic molecules [43], which justifies the increased antioxidant capacity of onion-based film samples with Laponite.

Onions exhibit an antioxidant capacity due to the presence of flavonoids and anthocyanins, which are classified as antioxidant compounds [44]. Therefore, to compare the antioxidant capacity of onion-based films and the actual increase in antioxidant capacity with the addition of Laponite, Figure 9c shows a comparison of onion-based films and PE films.

As expected, PE films do not show a significant antioxidant capacity (*p* < 0.05) and therefore the concentration of the fluorescent compound 2,5-DHB is higher when compared to onion-based films. The concentration of 2,5-DHB decreases by about 60% when compared with PE proving that pristine onion films have high antioxidant capacity. Moreover, the concentration of 2,5-DHB decreases further with the addition of Laponite to the onion-based films, reaching a concentration reduction of 82% compared to PE and 55% compared to the pristine films. These results prove that the addition of Laponite to onion-based films significantly (*p* < 0.05) increases the antioxidant capacity of the material due to its porous structure that acts as a radical scavenger.

However, as can be observed, with the increase in the Laponite concentration from 5% to 10% (*m*/*m*), there is no tendency to increase the antioxidant capacity, which may be related to the uneven saturation or dispersion of the compound in the onion-based films. Thus, it can be concluded that at low concentrations of the compound, there is a response to increase the antioxidant capacity.

According to the work of Soares et al. [13], onion film is recommended for preserving beef burgers, as it slows down the proliferation of unwanted microorganisms, stabilizes and improves color parameters and sensory attributes, as well as increasing overall consumer acceptance. With the addition of mineral clay, we propose that in addition to these benefits, the composite films can also offer additional antioxidant characteristics (according to UV-vis data and antioxidant tests) to the packaged products, increasing their shelf life.

## 3. Materials and Methods

### 3.1. Materials

For the film preparation, medium-sized yellow onion bulbs in commercial stages were acquired from markets in the city of São Carlos, São Paulo, Brazil. For the incorporation of clay, Laponite XLG (Na_0.7_[(Mg_5.5_Li_0.3_)Si_8_O_20_(OH)_4_]) obtained from BYK-Chemie was used for clay incorporation. Milli-Q^®^ water, sodium salicylate (CAS 54-21-7, 99.5%), 2,5-dihydroxybenzoic acid (CAS 490-79-9, 99%) from Sigma-Aldrich, HPLC-grade methanol (CAS 67-56-1), hydrogen peroxide (CAS 7722-84-1, 30%) and orthophosphoric acid (7664-38-2, 84%) from Scharlab were used for the antioxidant assay.

### 3.2. Onion Pulp Preparation and Clay Incorporation

The onion-based films were prepared according to the methodology of BioSmart Nanotecnologia Ltda. The dry mass was determined and then used for the incorporation of Laponite and subsequent preparation of the films by the casting method (DIAS et al.) [10]. The onion/Laponite films were obtained by varying the proportion of clay mass with the standardized 2% dry mass of onion pulp. The incorporation of the clay was performed considering 1%, 3%, 5%, and 10% proportions to the dry mass.

The Laponite dispersion was prepared using a 100 mL beaker with the addition of distilled water to a predetermined volume, which finally totaled 50 mL mixed with onion pulp. The Laponite solution was stirred in a magnetic stirrer for 10 min to obtain transparent dispersions.

Onion pulp was then added to the Laponite solution by stirring for 5 min to achieve complete dispersion. The final solution was then placed in a silicon-coated Petri dish and dried in an oven at 50 °C for 15 h. The samples were then named Pristine film, Laponite, Laponite 1%, Laponite 3%, Laponite 5%, and Laponite 10% according to the content of the clay mineral in the film.

### 3.3. Characterization of Pristine Onion and Laponite-Modified Films

#### 3.3.1. Physicochemical Properties

Pristine materials and all composites were analyzed in a D8-Advance X-ray diffractometer (Bruker). The analysis was performed by scanning the range 2θ from 5–70° in a continuous mode of 1 °/min, with a Soller divergence slit of 2.5° and a Lynxeye position-sensitive detector. To determine the crystallinity index, first, the separation of the crystalline peaks from the amorphous halo was first performed using the Gaussian function [45] expressed as follows:

(1) $$F\left(x_{i}\right)=\sum_{n=1}^{N}A_{n}G\left(x_{n},\;w_{n};\;x_{i}\right)+B(x_{i})\;\;$$

where *N* is the number of Gaussian peaks, $A_{n}$ is the peak area $G\left(x_{n},\;w_{n};\;x_{i}\right)$ is the Gaussian function and $B(x_{i})$ is the background in $x_{i}$. The term $G\left(x_{n},\;w_{n};\;x_{i}\right)$ can be expressed as

(2) $$G\left(x_{n},\;w_{n};\;x_{i}\right)=e^{-(x-n_{n}{)}^{2}/w_{n}}$$

where $x_{n}$ is the position of the peak and $w_{n}$ is the quantity related to the full width at half maximum (FWHM) of the nth peak.

The crystallinity index, CrI(%), was calculated by the expression:

(3) $$\mathrm{CrL}:\;\frac{A_{c}}{A_{c}+A_{a}}\times 100$$

where *A_c_* and *A_a_* are the areas of the crystalline and amorphous peaks.

The chemical structure of the bioplastics was analyzed using Fourier transform infrared (FTIR) spectroscopy in attenuated total reflection (ATR) mode in a Nicollet 6700 FTIR-Thermo Scientific spectrometer. Film spectra were recorded from 4000 to 400 cm^−1^, accumulating 64 scans with a resolution of 4 cm^−1^.

The morphology of the films was characterized by field emission scanning electron microscopy (FE-SEM; Carl Zeiss, model Supra 35-VP, Jena, Germany), operating at 2 kV.

Contact angle measurements were performed in a Ramé-Hart 260-F instrument, using deionized water and diiodomethane as probe liquids by the sessile drop method. For each sample, three drops were measured in duplicate for a total of six measurements per sample. To standardize the measurements, the measurement was taken 10 s after the drop.

The Young–Laplace theory was used to determine the hydrophilic/hydrophobic properties of the material, as well as the surface tension and adhesion properties of the surface. The surface tension was calculated using Equation (4), proposed by Wu [46], and the parameters of the probe liquids are listed in Table 2.

Equation (4) describes the calculation of surface tension for low-energy surface solid materials.

(4) $$\gamma_{\mathrm{Lv}}\;(1+\cos\;\theta )=4\;[\;\frac{\gamma_{SV}^{D}\gamma_{LV}^{D}}{\gamma_{SV}^{d}+\gamma_{LV}^{D}}+\frac{\gamma_{SV}^{P}\gamma_{LV}^{P}}{\gamma_{SV}^{P}+\gamma_{LV}^{P}}]$$

Here, γ*_L_* is the liquid probe tension, $\gamma_{L}^{d}$ and $\gamma_{L}^{p}$ are the dispersive and polar components of the liquid, and $\gamma_{s}^{d}$ e $\gamma_{s}^{p}$ are the dispersive and polar components of the solid, respectively. The sum of the polar and dispersive components of a solid results in its surface tension.

Absorbance and transmittance measurements in the UV-Vis region were obtained with a Perkin–Elmer spectrophotometer in the 200–800 nm range. From the absorbance spectra and the thickness of the films, the opacity was calculated according to Equation (5):

(5) $$\mathrm{Opacity}=\frac{{Abs}_{600\;(nm)}}{\;thickness\;(mm)}$$

#### 3.3.2. Water Vapor Permeability

The water vapor permeability of the film was determined using the ASTM E96-95 standard at a temperature of 25 °C. The weighing of the cups took place as follows: (1) on the first day, the mass was measured every 30 min for a total of 5 h and next (2) the weighing took place every 24 h for a total of 5 days.

The water vapor transmission rate (WVTR) was determined, using Equation (6), where α represents the mass of moisture permeated during this time (g h^−1)^ and A is the area of the sample available for permeation (m^2^).

(6) $$WVTR=\frac{\alpha }{A}$$

The WVP was calculated by the following expression, which also took into account the average film thickness (*e*):

(7) $$WVP=\frac{WVTR\times L}{∆P}$$

where, *L* is the film thickness ∆*P* is the difference in partial water vapor pressure across the film.

#### 3.3.3. Antioxidant Analysis

The equipment developed by Pezo et al. [47] was used for the antioxidant capacity assays of pristine and modified onion film samples with 5% and 10% Laponite.

The system scheme used consisted of a peristaltic pump with an injection flow rate set at 0.8 mL/min and a total air flow rate set at 3.76 L/min. The photoreactor used to generate the OH• radical from a 1.66% (*v*/*v*) hydrogen peroxide solution had a 300 mm of length by 30 mm of dyameter cylindrical quartz tube that was irradiated with ultraviolet light generated by eight 250 mm × 15 mm lamps (Philips UV fluorescent lamps, TL 8W/08 F8T5/BLB Hg, Eindhoven, The Netherlands) axially supported around the quartz tube.

Samples were placed in polyethylene (PE) bags according to the method described by Pezo et al. [47]. First, 1 dm^2^ of each sample was placed in 150 mm × 150 mm sealed bags, which were heat sealed and connected to the radical generator system OH•. Drechsler-type gas-washing vials filled with 50 g of 2 µg/g aqueous sodium salicylate solution were used to collect gases from the stream. Two replicates of each sample were analyzed and the samples nomenclature is described as follows: Polyethylene (PE), Onion film (Pristine film), Onion film with 5% (*m*/*m*) Laponite (Laponite 5%) and Onion film with 10% (*m*/*m*) (Laponite 10%). The samples with possible antioxidant capacity were compared with pristine onion films and PE, a polymer from a non-renewable source, and the oxidation of the samples was carried out for 24 h.

##### Analysis of the Reaction Product

The reaction between the OH• radicals and sodium salicylate (SA) present in the solution formed the product 2,5-dihydroxybenzoic acid (2,5-DHB), which is a fluorescent compound.

According to Figure 9a, after hydroxylation, the intermediate undergoes hydrogen abstraction by dissolved oxygen, resulting in the release of HO_2_ and a variety of oxidation by-products. The main by-products include 2,5-dihydroxybenzoic acid (2,5-DHB), 2,3-dihydroxybenzoic acid (2,3-DHB), and catechol, which are used as markers to determine the concentration of OH•. During the analyses, only 2,3-DHB and 2,5-DHB are employed, because catechol formation is negligible under the experimental conditions. In addition, the second isomer (2,3-DHB), a by-product of oxidation, is not considered due to its low fluorescence sensitivity [47].

The solution was injected into an HPLC equipped with a fluorescence detector. The chromatographic column was Atlantis TM dC18 3 µm (4.6 µm pore size, 100 mm length) and the mobile phase was acetate buffer (35 mmol/L; pH = 5.9): methanol (9:1). The temperature of the column and samples was 25 °C. The flow rate was set at 1 mL/min using isocratic mode and 10 µL injection volume. The wavelengths used were λ = 324 nm and λ = 448 nm.

As a result, the percentage of hydroxylation was calculated from the rule of three where 100% is the peak area of 2,5-DHB in the blank (pristine film) and x% is the peak area of 2,5-DHB in the sample with antioxidant (Laponite 5% and Laponite 10%).

Furthermore, due to the high fluorescence sensitivity, the reaction product was quantified using a pure 2,5-DHB standard. To accomplish this, the external calibration was plotted using different concentrations of the standard 2,5-DHB. In addition, the analytical parameters were determined and are listed in Table 3.

## 4. Conclusions

In this study, the potential antioxidant and UV protection properties of the onion/Laponite composite films have been proven. The X-ray analyses showed that with an increase in the Laponite content, there is an increase in crystallinity. However, the water vapor permeability values remained practically constant. This is associated with the presence of agglomerates in the structure of the material, as observed by SEM microscopy. In addition, their presence alters the wettability of the composite since an increase in the contact angle is observed with the presence of Laponite. The opacity of the films increased as a function of the Laponite content, with the highest values being observed for samples containing 5 and 10% Laponite. Furthermore, although the onion films were rich in flavonoids, the presence of Laponite had an additional effect of 82% on the antioxidant capacity of the films studied. Their structure and the presence of charges from sodium and magnesium ions can act as scavengers of radicals. These findings are highly significant as they suggest that the material has potential for use as biodegradable food packaging, capable of prolonging the shelf life of food products.

## Figures and Tables

**Table 1:** Crystallinity index, thickness, and opacity of pristine and composites films.

Film	Crl (%)	Thickness (μm)	Opacity (a.u/mm)
Pristine film	20.3	0.153 ± 0.02	7.7
Laponite 1%	17.5	0.152 ± 0.01	6.5
Laponite 3%	19.7	0.142 ± 0.01	7.5
Laponite 5%	25.5	0.149 ± 0.01	9.0
Laponite 10%	59.2	0.142 ± 0.02	9.1

**Table 2:** Parameters of the tension surface, dispersive and polar components for probe liquids, water, and diiodomethane.

Liquids		Water	Diiodomethane
Coef. Liq/vap	γ*_L_* (mN/m)	72.8	50.8
Polar Component Liq/Vap	$\gamma_{L}^{d}$(mN/m)	51.0	0.0
Dispersive Component	$\gamma_{L}^{p}$(mN/m)	21.8	50.8

**Table 3:** Analytical parameters of 2,5-DHB quantification method by HPLC-fluorescence.

Parameters	Value
Linearity range (µg/g)	0.0014–0.14
Correlation coefficient (r)	1.0000
Limit of Detection—LOD (ng/g)	0.41
Limit of Quantification—LOQ (ng/g)	13.75

## Data Availability

Relevant data can be made available by the authors on request.
